# How teachers’ student voice practices affect student engagement and achievement: exploring choice, receptivity, and responsiveness to student voice as moderators

**DOI:** 10.1007/s10833-024-09513-0

**Published:** 2024-07-22

**Authors:** Jerusha Conner, Dana L. Mitra, Samantha E. Holquist, Ashley Boat

**Affiliations:** 1 Department of Education, Villanova University, 302 St. Augustine Center, 800 Lancaster Ave. , 19085 Villanova, PA USA; 2 The Pennsylvania State University, 201 Old Main, University Park, 16802 PA USA; 3 Child Trends, 7315 Wisconsin Ave. Suite 1200 W, Bethesda, MD 20814 USA; 4 Search Institute, 3001 Broadway Street NE #310, Minneapolis, MN 55413 USA

**Keywords:** Student voice, Student engagement, Student agency, Student achievement, Choice

## Abstract

Strategies that promote student voice have long been championed as effective ways to enhance student engagement and learning; however, little quantitative research has studied the relationship between student voice practices (SVPs) and student outcomes at the classroom level. Drawing on survey data with 1,751 middle and high school students from one urban district, this study examined how the SVP of seeking students’ input and feedback related to their academic engagement, agency, attendance, and grades. Findings revealed strong associations between this SVP and student engagement. Additionally, results showed that having just one teacher who uses the SVP is associated with significantly greater agency, better math grades, higher grade point averages, and lower absent rates than having no teachers who do so. In models testing interaction effects with choice, responsiveness, and receptivity to student voice, teachers’ receptivity was strongly associated with all outcomes. Few interaction effects were found. This study contributes compelling evidence of the impact of classroom SVPs and teacher receptivity to student voice on desired student outcomes.

## Introduction

When he was Secretary of Education under President Obama, Arne Duncan observed, “Students know what’s working and not working in schools before anyone else” (Advocates for Children, 2012). Since the early 2000s, the notion that students have important insights into the effectiveness of their education and should therefore have a say in how to improve their experiences in schools and classrooms has become more widely accepted. Colloquially, this idea has come to be known as “student voice.” In practice, student voice refers to strategies and structures that enable students to have an influence on the educational decision-making that impacts their own and their peers’ experiences in school (Holquist et al., 2023). Several districts, schools, and individual teachers have introduced student voice practices (SVPs) in an effort to engage students and improve their learning (Biddle, 2017; Biddle & Huffnagel, 2019; Brasof, 2015; Giraldo-Garcia et al., 2020; Salisbury et al., 2019; Voight, 2015).

A large body of work attests to the benefits that accrue to students from participating in SVPs, including stronger leadership skills (Beaudoin, 2016; Lyons & Brasof, 2020) critical thinking and reflection (Geurts et al., 2023; Hipolito-Delgado et al., 2022), and communication skills (Bahou, 2012; Keogh & Whyte, 2005). Improved engagement, metacognition, and learning are also heralded in the literature as outcomes associated with student voice (Beattie & Rich, 2018; Geurts et al., 2023; Toshalis & Nakkula, 2012); however, these assertions about the gains students experience from SVPs have largely been derived from qualitative case study data or theory. The present study helps fill a gap in research on SVPs by using quantitative data to examine how students’ perceptions of their teachers’ use of classroom-level SVPs impact student outcomes, particularly their academic engagement, agency, attendance rates, and grades.

Theoretically, at the classroom level, student learning and achievement improve as a result of SVPs because student feedback, input, or involvement in decision-making informs and inspires teachers to change their instructional approach or classroom policies. These changes should not only result in more responsive teaching and more supportive learning environments but also help students to feel more invested in the learning experience. Knowing they have some level of influence on classroom decision-making can empower students as learners.

This theory, however, rests on many assumptions, not the least of which are that students will speak up when invited to do so and that teachers will listen and (know how to) adjust their practice accordingly. Qualitative research has found that this is not always the case (Black & Mayes, 2020; Thomson, 2011). When student perspectives are solicited, only to be seemingly ignored or discounted, students can become disengaged and demoralized (Mitra, 2018). Additionally, fearing that their ideas may be dismissed or misunderstood may lead some students to refrain from sharing ideas in the first place (Biddle & Huffnagel, 2019; Hipolito-Delgado, 2023; Silva, 2001).

SVPs may therefore be more effective when accompanied by two conditions: perceived “teacher receptivity” to student voice and perceived “teacher responsiveness” to student voice. This study explores the extent to which these two conditions moderate the relationship between SVPs and student outcomes. We also examine teachers’ provision of “choice” in the classroom as a potential moderator. Teachers who offer students choices in the classroom may be perceived by students as more flexible and adaptive in their teaching, more willing to tailor instruction, assessment, or curriculum to students’ needs and preferences, and therefore more likely to be receptive and responsive to student voice. Below, we review what is known about the relationship between student voice and student outcomes, the implementation of student voice at the classroom level, and the roles that choice, teacher receptivity, and teacher responsiveness play in facilitating student learning.

## Student voice and academic outcomes

Literature reviews on various types of student voice initiatives point to the positive academic outcomes associated with participating in student voice programs. These benefits include a stronger sense of agency and motivation as learners, a stronger sense of (disciplinary) identity, and improved student-teacher relationships (Geurts et al., 2023; Laux, 2018; Mercer-Mapstone et al., 2017).

Research also has documented associations between student voice and student engagement. In two separate studies, student voice (operationalized as student participation in decision-making) was found to be a significant predictor of both affective and cognitive engagement in school (Anderson, 2018; Zeldin et al., 2018). Studying a student voice initiative in a California school district serving predominantly low-income students of color, Voight and Velez (2018) found that students who participated showed improved school engagement relative to a matched comparison group who did not participate in the program. At the classroom level, one study using district-wide data found that when students felt their voice was listened to by their teachers, they reported higher affective engagement in their classes. This indicator of student voice was also indirectly linked to behavioral and cognitive engagement in class, through strengthened student-teacher relationships (Conner et al., 2022).

Because engagement has been well-established as an antecedent to learning and achievement (Lei et al., 2018), student voice scholars have connected SVPs to greater learning and achievement through this mechanism. It has been argued that by entrusting students with authority and valuing their expertise as learners, teachers who invite and use student voice promote students’ metacognition and ownership of their learning, thereby enhancing achievement outcomes (Beattie & Rich, 2018).

Some studies have found direct impacts of SVPs on learning and achievement outcomes. In their literature review of student participation in school and classroom decision-making, Mager and Nowalk (2012) identified 15 studies that found positive effects of participation on academic outcomes, such as “improved examination results, better academic performance or higher grades, greater student progress, and better goal attainment or student learning” (p. 44); however, the authors conclude that “too little methodologically strong research has been conducted” (p. 50), and the “low levels of evidence of better student academic achievement through participation in councils or in class decision-making” (p. 49) highlight a need for more high-quality research in this area. In Mercer-Mapstone and colleagues’ (2017) literature review of “pedagogical partnerships”–a particular type of student voice programming in which a student serves as a consultant to a teacher–, 19 studies attested to students’ improved content/disciplinary learning as a result of participation. One recent quantitative study drawing on panel data from Chicago showed that in schools that students described as more responsive to student voice, students had better grades and attendance patterns (Kahne et al., 2022). Other recent quantitative work, however, has turned up only limited evidence linking participation in student voice initiatives to improved achievement (Voight & Velez, 2018). To help substantiate claims from qualitative studies that student voice enhances students’ academic performance, more quantitative work is needed to clarify what kinds of SVPs lead to improved academic outcomes, for whom, and under what conditions.

The question of “for whom” is particularly important, given deep-seated educational inequities that continue to oppress low-income youth and youth of color. Student voice has been championed as a vehicle for educational equity, with its promise to help marginalized youth learn to critique the systems, policies, and practices that disadvantage them and advocate for change (Lac & Mansfield, 2018; Salisbury et al., 2020). Although initially “robust” student voice opportunities programs were “more likely to be in affluent, predominately White schools,” (Hipolito-Delgado et al., 2022, p. 2), they have since proliferated in schools and districts serving high proportions of low-income students of color (e.g. Bacca & Valladares, 2022; Giraldo-Garcia et al., 2020; Hipolito-Delgado et al., 2022; Ozer & Wright, 2012; Sussman, 2015; Taines, 2012; Zion, 2020). Most often, these programs involve a small (usually self-selecting) group of students either participating in a youth-participatory action research project or serving in an advisory capacity to adult decision-makers. Evidence is mounting that low-income students of color can and do derive important developmental benefits from participating in these opportunities (Hipolito-Delgado et al., 2022), and YPAR classes or after-school programs have been linked to some academic benefits, primarily through qualitative research (Anyon et al., 2018). Apart from studies on YPAR classes, research on how SVPs in the classroom affect the academic outcomes of students of color is sorely lacking.

## Student voice in the classroom

At the classroom level, SVPs give students a say in what is taught, how it is taught, how their learning is assessed, and/or what classroom norms or routines look like. These practices involve frank dialogue, reciprocal feedback, and an open exchange of ideas between the teacher and the learner.

Recent research has found that in the classroom, SVPs tend to take one of two forms: input/feedback or collaborative decision-making (Conner et al., 2024). Input and feedback involve the teacher asking for the students’ suggestions (input) or soliciting their constructive critique (feedback). The former is prospective, focused on what could be, while the latter is retrospective, attending to what was. Typically, teachers use surveys or group discussions to solicit input and feedback (Beaudoin, 2016; Conner, 2021).

The second form student voice can take in the classroom, collaborative decision-making, involves teachers partnering with their students to determine, evaluate, and ultimately select options for the class (Geurts et al., 2024). Collaborative decision-making may manifest as co-constructed lesson plans, co-constructed classroom rules, or co-created rubrics for assessing student work. It can also involve a class vote or “dot-mocracy,” in which students affix sticky dots to items to indicate their preferences from a list of mutually generated possibilities for curriculum or instructional activities (See Conner et al., 2024).

### Student voice versus choice in the classroom

Whether because they rhyme or because some scholars and practitioners believe they signify the same thing, the terms “student voice and choice” are often paired in the literature. Seiler (2011), for example, describes “a science curriculum model based on student voice and choice” (p. 362). Nasra (2021) describes how themes can be used in the English Language Arts curriculum “to encourage student choice and voice.” Despite their frequent conflation, the two sets of practices have key differences and are therefore best conceptualized as a Venn diagram, with some area of overlap. In choice, the teacher establishes the options and parameters and then gives students autonomy to choose among them. A three-by-three “choice board” in which students must complete three tasks in a column, a row, or along a diagonal is a paradigmatic example. Voice, by contrast, empowers students to generate possibilities for the classroom that teachers may never before have considered. In its ideal form, it invests students with influence and a greater degree of agency to shape their learning environment than choice.

While choice and voice can be conceptually distinguished, the two practices may be related. Choice has been found to be a pedagogical foundation of voice, meaning that developing comfort and facility with giving students choice in the classroom may actually build teachers’ capacity to engage in SVPs (Conner et al., 2024).

Given compelling research showing that choice in the classroom can promote student engagement and learning (Beymer et al., 2020; Patall et al., 2008, 2010; Schmidt et al., 2018), it is important to explore whether choice moderates or strengthens the relationship between student voice and student outcomes.

### Teacher receptivity and responsiveness to student voice

Responsiveness to student voice can be defined as taking action to address the concerns, critiques, recommendations, or ideas about educational practice and policy that students contribute. As mentioned above, recent research has found that responsiveness to student voice predicts key student outcomes, including greater attendance and achievement (Kahne et al., 2022). This research suggests that if students speak up to raise concerns or offer suggestions for improvement *and if* their teachers or administrators adjust their practice or policies in response, students benefit. Presumably the changes not only make schools more appealing places to be, promoting student attendance, but also make classroom teaching more effective, thereby enabling students to succeed academically. In a study with Australian primary students, Scarparolo & Mackinnon (2022) found that because teachers were responsive to students’ suggestions as they designed a unit of differentiated instruction, students reported greater engagement at the conclusion of the unit. Responsiveness to student voice seems to be a critical component of the process leading to better academic experiences and outcomes. In fact, writing about student voice in the classroom, McIntyre and colleagues (2005) assert that, “However good pupils’ ideas might be, it is teachers’ responsiveness to them that is ultimately important” (p. 151).

A less studied, but equally relevant phenomenon is perceived teacher receptivity to students’ ideas and student voice. Receptivity can be defined as a willingness to hear and consider student voice. Where responsiveness happens *after* a student engages in student voice, receptivity is a condition weighed *prior* to engaging. A student who believes his teachers or principal would not be receptive to his voice will be less likely to use it. For example, Taines (2012) quotes from several students who “believed it was pointless to engage in efforts to promote school change—even after their participation in the school activism program” because teachers and administrators “don’t care,’’ “don’t listen,” and ‘‘nothing else happens’’ as a result of raising their concerns:

Nikki held similar views of her school’s receptiveness. ‘‘Everybody’s not going to listen to me and say what I do.’’ In any event, ‘‘We’re already telling them and they’re not doing nothing,’’ Nikki said. On school-sponsored surveys, ‘‘They ask us, ‘Are you learning anything? Are your teachers helping you out? Do you feel safe?’. . I don’t see no change after you turn the survey in.’’ In fact, these students felt certain that if they tried to advocate for school change, they would personally suffer a backlash. One of Roland’s main school concerns was the cleanliness of the bathrooms. Asked if he could initiate improvement in this area, he replied, ‘‘They might snap on [get angry at] us then.” (p. 77).

Students may self-censor or refrain from student voice for several reasons, including fear of retaliation or fear of appearing disrespectful (Hipolito-Delgado, 2023). Some students, like Nikki quoted above, may use responsiveness (or lack thereof) to gauge receptivity.

While perceived receptivity has not been a focus of extant student voice research, scholars in this field do note its significance. In their study of the emotional politics of student voice, for instance, Black and Mayes (2020) reflect on how important it was for the student voice facilitators in the three schools they studied to describe their colleagues as “very keen” and “really open” (p. 1072) to student voice, implying that those who were receptive to student voice were more committed to putting students first than colleagues who were more ambivalent about student voice. In their study of a district-led student voice program, Giraldo-Garcia and colleagues (2020) found that “another factor that becomes critical for an effective program implementation is the institutional setting’s level of receptivity; if the program is not well-received, this may (and is likely to) result in poor implementation and will interfere with desired educational outcomes” (p. 54). Indeed, some research has found that teacher resistance to student voice (Biddle, 2019; Taines, 2014) or lack of readiness for student voice (Gillett-Swan & Sargeant, 2019) can undermine student voice from bringing about meaningful educational change. Believing their teachers will be receptive (rather than resistant or indifferent) to their concerns and ideas may well be a key precondition for SVPs to translate into better learning experiences; however, little work has examined how students’ perceptions of teachers’ receptivity to student voice shape the relationship between SVPs in the classroom and student outcomes.

## The present study

The present study investigates SVPs in relation to student outcomes, focusing on how many of their teachers students felt engaged in SVPs in the classroom. Drawing on survey data from students attending two middle schools and two high schools in a single district, we examine the following research questions:

1) How are students’ perceptions of their teachers’ use of SVPs associated with student academic engagement, agency, attendance, and grades?

2) Do student perceptions of teachers’ receptivity to their ideas, provision of choice in the classroom, and responsiveness to student voice moderate the relationships between classroom-level SVPs and student outcomes?

Guided by extant research and theory, we hypothesized that we would find that more teachers using SVPs would be related to stronger engagement, agency, attendance, and grades. In particular, we expected to see higher overall GPAs and higher English Language Arts (ELA) grades, but not necessarily higher Math grades. In response to our second research question, we expected that choice, responsiveness, and receptivity would each strengthen the relationships between SVPs and the aforementioned outcomes.

## Method

### Procedures

The current study uses data from a survey designed to assess SVPs. The survey was administered by four partner schools (two middle schools and two high schools) using standardized administration procedures in winter 2023. School partners sent a parent-opt out form to all students’ parents/guardians about one week prior to survey administration. The form was sent in both English and Spanish. School partners invited all current students to take the survey. The survey was administered online in English via a secure data collection platform and took about 15–20 min to complete. It was made clear to participants that the survey was anonymous, participation was completely voluntary, and that choosing not to participate would in no way impact students’ relationship with their school. Teachers within the four schools were asked to administer the survey during dedicated class time to communicate the importance of the survey and encourage students to take the survey seriously. Students did not receive incentives for completing the survey.

### Participants

The study was conducted in a large urban district, known for its commitment to student voice. Located in the Western United States, the district serves approximately 65,000 students from Pre-K to 12th grade. Student voice features prominently in the district’s Strategic Plan, and the district has hired Student Voice liaisons at both the district and the school levels. District administrators selected the four schools in which to conduct this research based on the schools’ emerging or ongoing work to amplify student voice and their principals’ commitment to student voice.

A total of 1,751 students from four schools were included in the current study. Response rates across the four schools ranged from 10 to 58%. Of the respondents, 51% were middle school students and 49% were in high school. About half of the participants identified as female (49.1%), 47.2% identified as male, and 3.7% identified with another gender (e.g., non-binary). Additionally, 4.9% of students identified as transgender. Students predominantly identified as Hispanic/Latiné (66.5%), 11.4% identified as White, 8.5% identified as Multiracial, 4.7% identified as Asian/Pacific Islander, 3.5% identified as Black/African American, 1.5% identified as American Indian/Native American, and 2.1% identified as another race/ethnicity. A little less than half of students reported experiencing no family financial strain (48.5%), while 20.2% reported experiencing some strain, and 11.3% reported experiencing a lot of strain.

### Measures

#### Student demographics

Students reported on their demographic information, including their grade level (ranging 6th-12th grade), gender identity, race/ethnicity, and family financial strain. Gender was measured using a dummy coded variable (*girls* = 1 and *boys* = 0). Participants who identified as non-binary or self-described their gender were excluded from the analytic analyses due to the small sample size (*n* = 63). Over half of the student population identified as Hispanic or Latiné. Due to the small sample size of other racial and ethnic groups, we created dummy coded variables to capture students who identified as Hispanic/Latiné, White, and Non-Hispanic/Latiné students of color. Students who identified as Hispanic/Latiné served as the reference group. All students in the school district receive free and reduced lunch regardless of family income level. Therefore, students were asked to respond to a question regarding their family’s financial strain as a proxy for student socioeconomic status. Family financial strain was assessed through the following prompt: “Which of the following statements best describes your family’s financial situation?” Students responded to the prompt using the following response options: *we cannot buy the things we need sometimes* = 2; *we have just enough money for the things we need* = 1; *we have no problem buying the things we need* = 0. Higher scores represent more family financial strain.

#### English language learner services

Schools provided administrative data on all students who participated in the survey, including whether or not students were currently receiving any English language learner (ELL) services. ELL services were measured using a dummy coded variable (*yes* = 1 and *no* = 0).

#### School site

The four school sites were controlled for in analysis using dummy coded variables to represent each school. One of the middle schools served as the reference group (*n* = 335).

#### Classroom-level student voice practices

One dimension of classroom-level SVPs, seeking student input/feedback, was used in this analysis. Seeking input/feedback was assessed with an eight-item scale (please see Conner et al., 2023 for scale validation). Students were asked to respond to the following prompt, “How many of your teachers do the following?” Example items then included: “ask students what they want to learn about in the class,” “ask for students’ ideas about how to make the classroom better,” and “ask for students’ suggestions about how they can get better at teaching.” All items were assessed on a 4-point scale ranging from *None* (0) to *Most*,* more than half of my teachers* (3). All items were used to create a mean score. The reliability for this scale was strong (α = 0.90).

#### Choice

Students were asked about their perceptions of their teachers’ provision of choice within classrooms through a five-item scale. Items include: “allow students to choose their own topics for projects or assignments,” “let students choose the types of assignment they work on (for example, group work, games),” “give students choices for which tasks to complete for homework,” and “allow students to choose how they want to work in the classroom (for example, with a partner, with a group, alone).” Students were asked to respond to the following prompt, “How many of your teachers do the following?” Items were assessed on a 4-point scale ranging from *None* (0) to *Most*,* more than half of my teachers* (3). All items were used to create a mean score. The reliability for this scale was strong (α = 0.82).

#### Teacher receptivity

Students were asked about teacher receptivity through a three-item scale. Items include: “how many of your teachers would you feel comfortable going to with an idea about how to make their class better?” “how many of your teachers would you feel comfortable approaching if you had a concern about the classroom?” and “if you made a suggestion to a teacher, how many of them would take your ideas seriously?” Students were asked to respond to the following prompt, “How many of your teachers do the following?” Items were assessed on a 4-point scale ranging from *None* (0) to *Most*,* more than half of my teachers* (3). All items were used to create a mean score. The reliability for this item was strong (α = 0.84).

#### Teacher responsiveness

Students were asked about teacher responsiveness through a four-item scale. Students responded to the following prompt, “You indicated one or more of your teachers ask students questions about their experiences as learners. How do those teachers respond to the ideas students share?” Students then responded to the following items: “those teachers actually listen to students’ answers,” “those teachers take students’ answers and use them,” “those teachers use students’ answers to make the classroom better,” and “those teachers tell us how students’ answers were used to make the classroom better.” Items were assessed on a 4-point scale ranging from *Not at all or a little like those teachers* (1) to *Extremely like those teachers* (4). All items were used to create a mean score. The reliability for this scale was strong (α = 0.81).

#### Academic engagement

Academic engagement was assessed using a previously validated, widely-used nine-item measure from the Stanford Survey of Adolescent School Experiences (Pope et al., 2015), which taps affective, behavioral, and cognitive engagement (Fredricks et al., 2004). All items began with the stem, “How often do you.” Example items include “complete your school assignments,” “have fun in your classes,” “find value in what you do in your classes,” and “think your schoolwork helps you to deepen your understanding or improve your skills.” Items were assessed on a 4-point scale ranging from *Never* (1) to *Often* (4). The reliability for this scale was strong (α = 0.91).

#### Student voice agency

Students were asked about student voice agency using a three-item measure. Items include: “I give ideas to school leaders about how to improve the school when I am asked,” “I give ideas to school leaders about how to improve the school, even when I am not asked,” and “I have participated in at least one of the opportunities available at school to share my ideas about how to improve our school.” Items were assessed on a 4-point scale ranging from *Strongly Disagree* (1) to *Strongly Agree* (4). All items were used to create a mean score. The reliability for the scale was moderate (α = 0.74).

#### Absent rate

Schools also provided an absent rate for each student. Absent rate was calculated by taking the total number of days absent divided by the total number of possible school days. Absent rate ranged from 0.0 to 0.85 (*M* = 0.11; *SD* = 0.12).

#### Grades

We included two different measures of student grades. First, students reported on their math grade and their ELA grade. Students were asked, “This school year, what grades do you typically get in your [math class/ELA class]?” The question prompt was adjusted to ask specifically about either math class or ELA class. Response options across both question prompts ranged from *Mostly Fs* (1) to *Mostly As* (5). Schools provided administrative data for all students who participated in the survey including students’ most recent cumulative GPA. GPAs ranged from 0.0 to 4.0 (*M* = 2.73; *SD* = 0.85).

The rationale for including these disparate measures of achievement stemmed partially from the research base. Other studies of the effect of student voice on student achievement using GPA have turned up mixed outcomes (Kahne et al., 2022; Voight & Velez, 2018). Including GPA allows us to contribute to these conversations. Additionally, studies have shown how student voice can help students improve their critical analysis and communication skills (Bahou, 2012; Hipolito-Delgado et al., 2022; Keogh & Whyte, 2005), skills that are particularly relevant in the English classroom. Therefore, examining ELA grades, separate from GPA, was warranted. Math was included as a check or counter-balance to ELA. In addition, we believed it was important to balance self-reported academic achievement with administrative data. Although some studies dispute the credibility of student self-reported grades (Kuncel et al., 2005), other research concludes that students can provide accurate and valid indicators of their performance (Wigfield & Wagner, 2005). Therefore, we thought it would be prudent to include data from both sources.

### Analytic strategy

Preliminary analyses examined descriptive statistics, multicollinearity, and intercorrelations between study variables. Subsequently, we examined whether academic engagement, student voice agency, attendance, and achievement outcomes varied among students who reported varying levels of the number of teachers who used the classroom-level SVP, seeking input and feedback. IBM SPSS software (version 29.0.1) was used to run descriptives, multicollinearity diagnostics, bivariate correlations, and a one-way ANOVA to examine the differences in average levels of student academic engagement, agency, attendance, and performance by the number of teachers using SVPs. Tukey post hoc tests were applied to assess any statistically significant differences among groups.

To examine the association between the number of teachers using classroom SVPs by seeking student input/feedback and student outcomes as moderated by students’ perceptions of their teachers’ receptivity, responsiveness, and provision of choice, we conducted a series of regression models. We first examined whether students’ report of teachers’ use of seeking input/feedback was associated with student engagement, student voice agency, absent rate, and academic performance while controlling for student gender, race/ethnicity, grade level, ELL services, family financial strain, and school site. For each model, a quadratic term of classroom SVPs was assessed in order to determine if SVPs demonstrated a linear or curvilinear relationship with each outcome. In these models, the linear and quadratic terms were centered at the mean. If the quadratic term was statistically significant, then it was retained in subsequent models.

We then specified regression models where the number of teachers using classroom SVPs was included as an independent variable, while academic engagement, student voice agency, absent rate, and academic performance variables served as dependent variables, and teachers’ provision of choice, teacher responsiveness, teacher receptivity, family financial strain, gender identity, race/ethnicity, grade level, ELL services, and school site served as control variables. Interaction models were then specified to examine the provision of choice, teacher receptivity, and teacher responsiveness as moderators. Interaction terms between the classroom SVP variable (including both linear and quadratic terms, where appropriate) and choice, teacher receptivity, and teacher responsiveness were added to the respective main effects models to test the moderating role of each construct. All variables were standardized prior to the creation of interaction terms. We used regions of significance, known as the Johnson–Neyman technique, to evaluate the interaction (Preacher et al., 2007). This method defines regions of significance on the moderator and represents the range of moderator values at which the simple slope of the outcome on the predictor is significantly different from zero. All analyses were completed using Mplus version 8.8. Full-information maximum likelihood estimation was used to improve estimation under conditions of missing data (Enders, 2022).

## Results

### Descriptive statistics and intercorrelations

Descriptive statistics and bivariate correlations of the main variables of interest can be found in Table 1. Bivariate correlations show that SVPs were positively correlated with teacher provision of choice (*r* = .76, *p* < .001), receptivity (*r* = .57, *p* < .001), and responsiveness (*r* = .50, *p* < .001). SVPs were also positively correlated with student outcomes including academic engagement (*r* = .36, *p* < .001), student voice agency (*r* = .27, *p* < .001) and ELA grade (*r* = .06, *p* < .05). SVPs were negatively correlated with absenteeism rate (*r* = − .11, *p* < .001), but unrelated to Math grades and unweighted GPA. The variance inflation factor (VIF) was used to examine the potential for multicollinearity among independent variables. All variables had VIF values less than 3.0. VIF values above 3.0 are regarded as indicating multicollinearity (Hair et al., 2019). The most extreme VIF value among the independent variables was 2.32, which was under the suggested value, indicating no multicollinearity issues.

**Table 1:** Means, standard deviations, and intercorrelations between study variables

Variable	1	2	3	4	5	6	7	8	9	10	11	12	13	14	15
1. Female															
2. Latine	0.09***														
3. Grade level	− 0.004	− 0.04													
4. ELL	− 0.06*	0.21***	0.002												
5. FSS	− 0.03	0.06*	− 0.03	− 0.05											
6. SVPs	0.01	0.04	− 0.04	0.07**	0.11***										
7. Choice	0.002	0.04	− 0.08**	0.08**	0.07*	0.76***									
8. Receptivity	− 0.04	− 0.07**	0.02	− 0.04	0.07*	0.57***	0.51***								
9. Responsive	− 0.01	− 0.01	0.08	0.04	0.05	0.50***	0.40***	0.43***							
10. AE	0.10***	− 0.02	− 0.02	0.01	0.06*	0.36***	0.32***	0.40***	0.23***						
11. Agency	0.02	− 0.03	0.02	0.05	0.04	0.27***	0.32***	0.23***	0.14**	0.22***					
12. Absent rate	0.05*	0.03	0.20***	0.09***	− 0.10***	− 0.11***	− 0.09***	− 0.15***	− 0.12**	− 0.19***	− 0.07*				
13. Math grade	− 0.03	− 0.14***	− 0.07**	− 0.23***	0.09**	0.03	0.02	0.11***	0.06	0.22***	− 0.02	− 0.32***			
14. ELA grade	0.08**	− 0.09**	0.01	− 0.18***	0.05	0.06*	0.04	0.13***	0.08	0.25***	0.07	− 0.34***	0.42***		
15. GPA	0.10***	− 0.11***	− 0.02	− 0.27***	0.10***	0.03	0.02	0.15***	0.07	0.23***	0.07*	− 0.53***	0.61***	0.54***	
Mean	0.49	0.67	8.59	0.23	1.37	1.38	1.38	1.65	2.51	2.83	2.24	0.11	3.42	4.00	2.73
SD	0.50	0.47	1.86	0.42	0.68	0.85	0.81	0.89	0.67	0.67	0.64	0.12	1.28	1.07	0.84

Note. FFS = Family Financial Strain; SVPs = Student Voice Practices; AE = Academic Engagement. * *p* < .05; ** *p* < .01; *** *p* < .001

### One-way ANOVA

Table 2 presents the one-way ANOVA results. Four groups were made based on thresholds aligned with the number of teachers students reported using classroom-level SVPs: zero teachers (16%), one teacher (36.3%), some, less than half of teachers (35.1%), and most, more than half of teachers (12.6%). Mean levels of student academic engagement significantly differed among all four groups, with increasing levels of engagement and agency as students reported more teachers using classroom SVPs. There were also significant differences in student absent rate. Students who reported increasing numbers of their teachers using classroom SVPs reported lower absent rates relative to students who reported that none of their teachers used classroom SVPs.

**Table 2:** Academic engagement and performance outcomes among students who report varying numbers of teachers using classroom SVPs

	Academic Engagement Mean (SD)	Student Voice Agency Mean (SD)	Absent Rate Mean (SD)	ELA Grade Mean (SD)	Math Grade Mean (SD)	Unweighted GPA Mean (SD)
0 Teachers (16%)	2.40 (0.70)	2.00 (0.60)	0.14 (0.14)	3.86 (1.23)	3.25 (1.35)	2.61 (0.88)
1 Teacher (36.3%)	2.77 (0.60) ^a^	2.16 (0.63) ^a^	0.11 (0.12) ^a^	4.04 (1.06)	3.48 (1.30)	2.83 (0.86) ^a^
Some (less than half) Teachers (35.1%)	2.95 (0.62) ^a, b^	2.30 (0.56) ^a, b^	0.11 (0.12) ^a^	4.02 (1.02)	3.52 (1.21) ^a^	2.81 (0.80) ^a^
Most (more than half) Teachers (12.6%)	3.20 (0.69) ^a, b, c^	2.63 (0.69) ^a, b, c^	0.09 (0.08) ^a^	4.10 (0.99)	3.33 (1.35)	2.72 (0.90)
*F*	63.17***	18.06***	6.53 ***	2.10	3.06*	4.63**

Note. * *p* < .05, ** *p* < .01, *** *p* < .001

^a^ = mean score significantly differs from 0 teachers seeking input/feedback

^b^ = mean score significantly differs from 1 teacher seeking input/feedback

^c^ = mean score significantly differs from some teachers seeking input/feedback

Significant differences also emerged in mean GPA, such that students who reported at least one teacher used classroom SVPs (*M* = 2.83; *SD* = 0.86) and students who reported some teachers used classroom SVPs (*M* = 2.81; *SD* = 0.80) reported higher GPAs than students who reported none of their teachers used SVPs (*M* = 2.61; *SD* = 0.88). There were no significant differences in students’ self-reported ELA grades by the four groups. Finally, there was a significant difference in mean self-reported math grades, such that students who reported that some of their teachers used classroom SVPs reported higher math grades (*M* = 3.52; *SD* = 1.21) than students who reported none of their teachers used classroom SVPs (*M* = 3.25; *SD* = 1.35).^1^

### Main effect models

Tables 3 and 4 present both main effect and moderation regression models. The number of teachers using classroom SVPs was associated with greater academic engagement (linear β = 0.18, *SE* = 0.02, *p* < .001; quadratic β = 0.16, SE = 0.02, *p* < .001), greater student voice agency (linear β = 0.20, *SE* = 0.03, *p* < .001; quadratic β = 0.18, SE = 0.02, *p* < .001), higher self-reported ELA grades (linear β = 0.06, *SE* = 0.03, *p* < .05), and lower absent rates (linear β = − 0.10, *SE* = 0.02, *p* < .001), while controlling for student characteristics. The significant quadratic terms found in the academic engagement, student voice agency, and unweighted GPA models suggest a significant curvilinear trend. The quadratic term was not statistically significant in the absent rate, ELA grade, or math grade models. Therefore, these models were specified with the quadratic term removed for a more parsimonious model.

**Table 3:** Provision of choice, teacher receptivity, and responsiveness as moderators of the relationship between student voice practices (SVPs) and student engagement, student voice agency and absent rate

	Academic Engagement			Student Voice Agency			AbsentRate^a^		
	β (*SE*)	β (*SE*)	β (*SE*)	β (*SE*)	β (*SE*)	β (*SE*)	β (*SE*)	β (*SE*)	β (*SE*)
Female	0.10 (0.03)***	0.10 (0.02)***	0.11 (0.02)***	0.05 (0.04)	0.05 (0.04)	0.06 (0.04)	0.05 (0.02)*	0.05 (0.02)*	0.05 (0.02)*
Non-Latiné student of color	0.06 (0.03)*	0.06 (0.03)*	0.06 (0.03)*	0.05 (0.04)	0.05 (0.04)	0.05 (0.04)	− 0.06 (0.02)*	− 0.06 (0.02)*	− 0.06 (0.02)*
White	− 0.01 (0.03)	− 0.03 (0.03)	− 0.03 (0.03)	0.02 (0.04)	0.01 (0.04)	0.02 (0.04)	0.04 (0.03)	0.05 (0.03)	0.05 (0.03)
Grade level	0.04 (0.05)	0.01 (0.05)	− 0.01 (0.05)	0.04 (0.06)	0.03 (0.06)	0.04 (0.06)	0.10 (0.05)*	0.12 (0.05)**	0.12 (0.05)**
ELL services	0.01 (0.03)	0.01 (0.02)	0.01 (0.02)	0.06 (0.04)	0.06 (0.04)	0.06 (0.04)	0.09 (0.02)***	0.09 (0.02)***	0.09 (0.02)**
FSS	− 0.04 (0.03)	− 0.02 (0.03)	− 0.03 (0.03)	− 0.04 (0.04)	− 0.04 (0.04)	− 0.04 (0.04)	0.08 (0.03)**	0.08 (0.03)**	0.08 (0.03)**
Linear SVPs	0.18 (0.02)***	0.19 (0.04)***	0.08 (0.04)*	0.20 (0.03)***	0.06 (0.06)	0.02 (0.05)	− 0.10 (0.02)***	− 0.01 (0.04)	0.01 (0.04)
Quadratic SVPs	0.16 (0.02)***	− 0.05 (0.03)*	0.07 (0.03)*	0.18 (0.02)***	0.09 (0.04)*	0.02 (0.05)			
Choice		0.07 (0.04)*	0.02 (0.04)		0.25 (0.06)***	0.26 (0.06)***		0.01 (0.04)	0.01 (0.04)
Receptivity		0.22 (0.03)***	0.20 (0.04)***		0.09 (0.04)*	0.04 (0.06)		− 0.10 (0.03)***	− 0.09 (0.03)**
Responsiveness		0.04 (0.05)	0.10 (0.07)		− 0.01 (0.05)	− 0.14 (0.06)*		− 0.08 (0.04)	− 0.09 (0.04)
Linear SVPs X Choice			− 0.10 (0.04)*			0.08 (0.05)			− 0.01 (0.04)
Quadratic SVPs X Choice			0.18 (0.07)*			− 0.11 (0.09)			
Linear SVPs X Receptivity			0.05 (0.03)			0.01 (0.05)			0.01 (0.03)
Quadratic SVPs X Receptivity			0.01 (0.05)			0.06 (0.04)			
Linear SVPsX Responsiveness			− 0.12 (0.06)*			− 0.07 (0.06)			0.05 (0.05)
Quadratic SVPs X Responsiveness			− 0.06 (0.09)			0.29 (0.10)**			
*R* ^2^	0.15***	0.20 ***	0.21 ***	0.10 ***	0.15 ***	0.17 ***	0.09 ***	0.10***	0.10***

Note. * *p* < .05, ** *p* < .01, *** *p* < .001; Standardized coefficients are reported; School sites were coded as dummy variables and were controlled for in analyses. Latiné students served as the reference category. ELL = English language learner; FFS = Family financial strain. ^a^ No significant quadratic effects were found in the absent rate model, thus the quadratic term was removed for a more parsimonious model

**Table 4:** Provision of choice, teacher receptivity, and responsiveness as moderators of the relationship between SVPs and student self-reported grades and GPA

	Math Grade^a^			ELA Grade^a^			Unweighted GPA		
	β (*SE*)	β (*SE*)	β (*SE*)	β (*SE*)	β (*SE*)	β (*SE*)	β (*SE*)	β (*SE*)	β (*SE*)
Female	− 0.03 (0.03)	− 0.03 (0.03)	− 0.03 (0.03)	0.07 (0.03)**	0.08 (0.03)**	0.07 (0.03)**	0.09 (0.02)***	0.09 (0.02)***	0.08 (0.03)**
Non-Latiné; student of color	0.08 (0.03)**	0.08 (0.03)**	0.08 (0.03)**	0.07 (0.03)**	0.07 (0.03)*	0.07 (0.03)**	0.07 (0.02)**	0.06 (0.02)*	0.09 (0.03)**
White	0.06 (0.03)*	0.05 (0.03)*	0.05 (0.03)*	0.01 (0.03)	0.002 (0.03)	0.003 (0.03)	0.04 (0.02)	0.03 (0.02)	0.02 (0.03)
Grade level	0.19 (0.05)***	0.18 (0.05)***	0.17 (0.05)***	0.20 (0.05)***	0.19 (0.05)***	0.19 (0.05)***	0.25 (0.04)***	0.23 (0.04)***	0.19 (0.05)***
ELL services	− 0.22 (0.02)***	− 0.22 (0.03)***	− 0.22 (0.02)***	− 0.17 (0.03)***	− 0.17 (0.03)***	− 0.17 (0.03)***	− 0.24 (0.02)***	− 0.23 (0.02)***	− 0.20 (0.03)***
FFS	− 0.09 (0.03)**	− 0.08 (0.03)**	− 0.09 (0.03)**	− 0.04 (0.03)	− 0.04 (0.03)	− 0.04 (0.03)	− 0.09 (0.03)***	− 0.09 (0.03)***	− 0.09 (0.03)**
Linear SVPs	0.03 (0.03)	− 0.01 (0.04)	0.01 (0.04)	0.06 (0.03)*	0.001 (0.05)	0.01 (0.05)	0.04 (0.02)	− 0.04 (0.04)	− 0.05 (0.05)
Quadratic SVPs							− 0.09 (0.02)***	− 0.09 (0.02)***	0.05 (0.05)
Choice		− 0.03 (0.04)	− 0.02 (0.04)		− 0.03 (0.04)	− 0.03 (0.04)		− 0.03 (0.04)	− 0.08 (0.05)
Receptivity		0.09 (0.03)**	0.08 (0.03)*		0.11 (0.03)***	0.11 (0.03)***		0.14 (0.03)***	0.16 (0.04)***
Responsiveness		0.05 (0.05)	0.06 (0.05)		0.05 (0.05)	0.04 (0.05)		0.07 (0.05)	0.09 (0.07)
Linear SVPs X Choice			− 0.02 (0.04)			0.01 (0.04)			− 0.09 (0.05)
Quadratic SVPs X Choice									0.16 (0.07)*
Linear SVPs X Receptivity			0.004 (0.03)			− 0.04 (0.03)			− 0.06 (0.03)
Quadratic SVPs X Receptivity									− 0.08 (0.06)
Linear SVPs X Responsiveness			− 0.11 (0.05)*			− 0.01 (0.05)			0.02 (0.06)
Quadratic SVPs X Responsiveness									− 0.05 (0.09)
*R* ^2^	0.12***	0.13***	0.14***	0.08***	0.09***	0.10***	0.14***	0.16***	0.17***

Note. * *p* < .05, ** *p* < .01, *** *p* < .001; Standardized coefficients are reported; School sites were coded as dummy variables and were controlled for in analyses. Latiné students served as the reference category. ELL = English language learner; FFS = Family financial strain. ^a^ No significant quadratic effects were found in the ELA or math models, thus the quadratic term was removed for a more parsimonious model

Once students report of teachers’ provision of choice, teacher responsiveness, and teacher receptivity were included in the models, classroom SVPs remained significantly associated with greater academic engagement only (linear β = 0.19, *SE* = 0.04, *p* < .001; quadratic β = − 0.05, SE = 0.03, *p* < .05). The number of teachers using classroom SVPs was not significantly associated with greater student voice agency, lower absent rate, or higher academic performance outcomes. The significant quadratic terms found in the student voice agency and unweighted GPA models remained with the inclusion of teachers’ provision of choice, teacher responsiveness, and teacher receptivity, suggesting a curvilinear relationship.

Students’ experiences of their teachers’ responsiveness were unrelated to all student outcomes. Students’ experience of their teachers’ provision of choice was significantly associated with greater student voice agency only (β = 0.25, *SE* = 0.06, *p* < .001). In contrast, teacher receptivity was associated with all academic outcomes including greater academic engagement (β = 0.21, *SE* = 0.03, *p* < .001), greater student voice agency (β = 0.09, *SE* = 0.04, *p* < .05), higher self-reported ELA grade (β = 0.11, *SE* = 0.03, *p* < .001), higher self-reported math grade (β = 0.09, *SE* = 0.03, *p* < .01), higher GPA (β = 0.16, *SE* = 0.04, *p* < .001), and a lower absent rate (β = − 0.10, *SE* = 0.03, *p* < .001).

### Moderation models

Teacher receptivity did not significantly interact with classroom SVPs across any of the models. Only teachers’ provision of choice and teacher responsiveness significantly interacted with classroom SVPs.

**Fig. 1:**
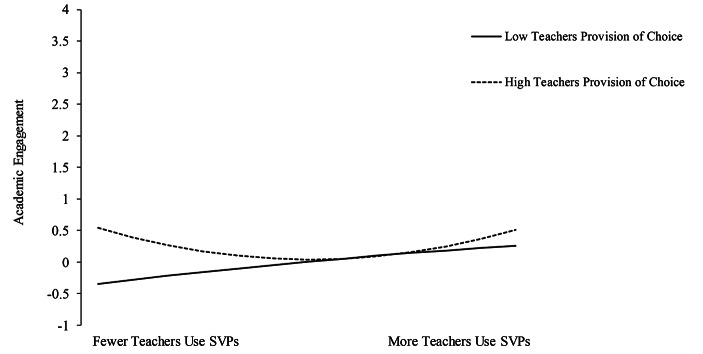
Simple slope analyses of the interaction between SVPs and choice on student academic engagement

The interaction between linear and quadratic classroom SVPs and teacher provision of choice was significantly associated with student academic engagement (linear β = − 0.10, SE = 0.04, *p* < .05; quadratic β = 0.18, SE = 0.07, *p* < .05). Figure 1 shows there is a curvilinear relationship, among students reporting more teachers providing choice, such that students reported greater academic engagement both when reporting fewer teachers using SVPs and more teachers using SVPs. Among students reporting fewer teachers providing choice, there appears to be a more linear relationship such that students report greater academic engagement when also reporting more teachers using SVPs.

**Fig. 2:**
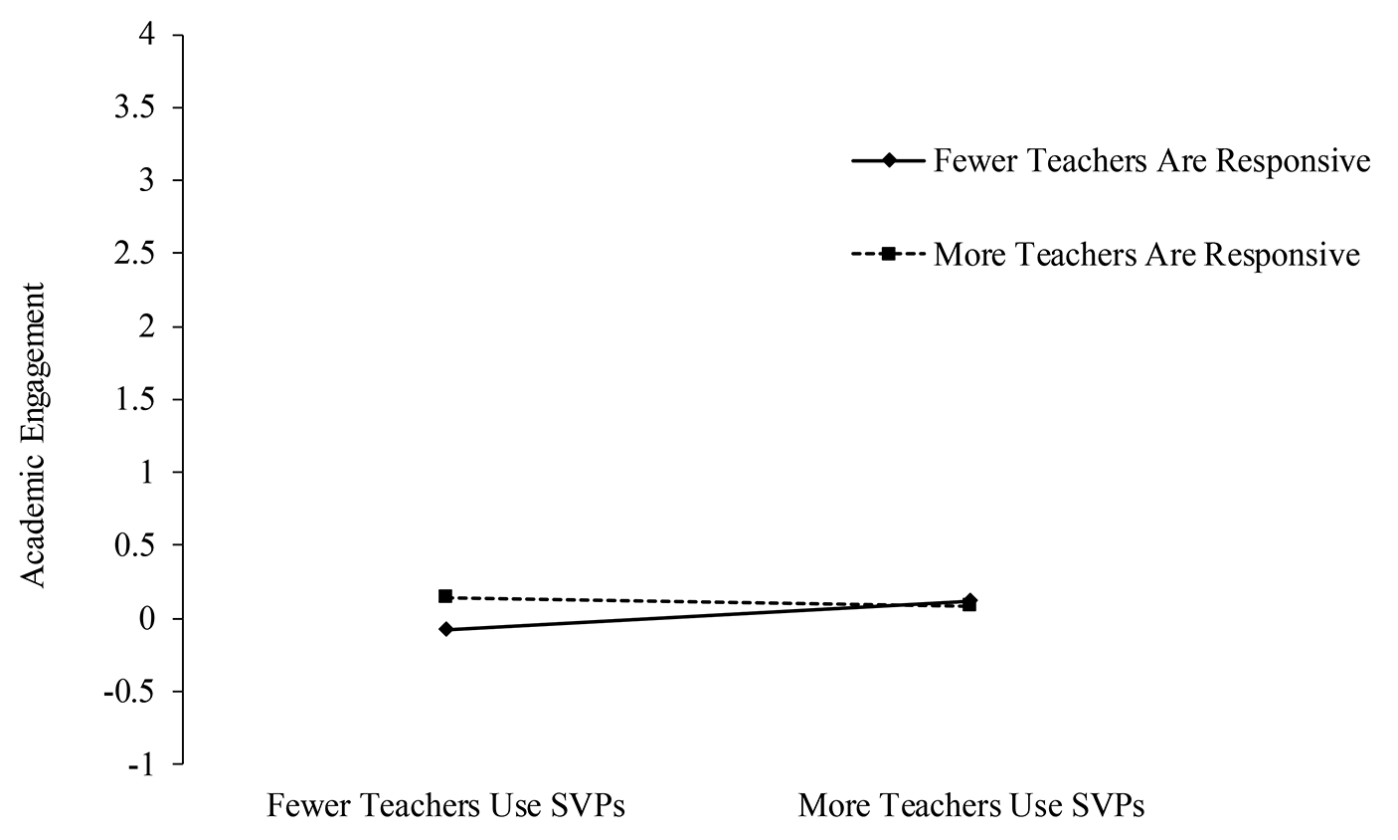
Simple slope analysis of the interaction between SVPs and responsiveness on student academic engagement

The interaction between linear classroom SVPs and teacher responsiveness was also significant (linear β = − 0.12, SE = 0.06, *p* < .05). Figure 2 shows that the benefit of teacher responsiveness on academic engagement was limited to students who reported fewer teachers using classroom SVPs. This pattern was confirmed by examining simple slopes. A greater likelihood of teachers being responsive was related to significantly higher engagement for those students reporting fewer teachers using SVPs (simple slope at 1 SD below the mean: β = 0.20, SE = 0.07, *p* < .01), but there was no statistically significant impact among students who reported more teachers using SVPs (simple slope at 1 SD above the mean: β = − 0.01, SE = 0.05, *p* = ns).

**Fig. 3:**
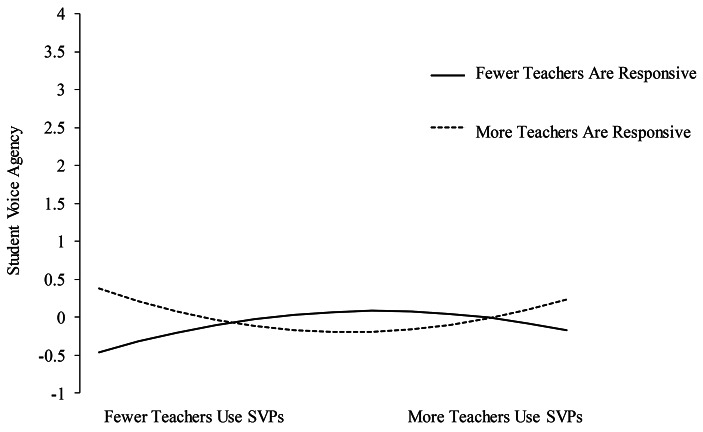
Simple slope analysis of the interaction between quadratic SVPs and responsiveness on student agency

The interaction term between quadratic SVPs and teacher responsiveness was significantly associated with student voice agency (β = 0.29, SE = 0.10, *p* < .01). Figure 3 shows there is a curvilinear relationship. Among students reporting more teachers as responsive, students reported greater agency both when reporting fewer teachers using SVPs and more teachers using SVPs. In contrast, among students reporting fewer teachers as responsive, students reported lower agency both when reporting fewer teachers using SVPs and more teachers using SVPs.

**Fig. 4:**
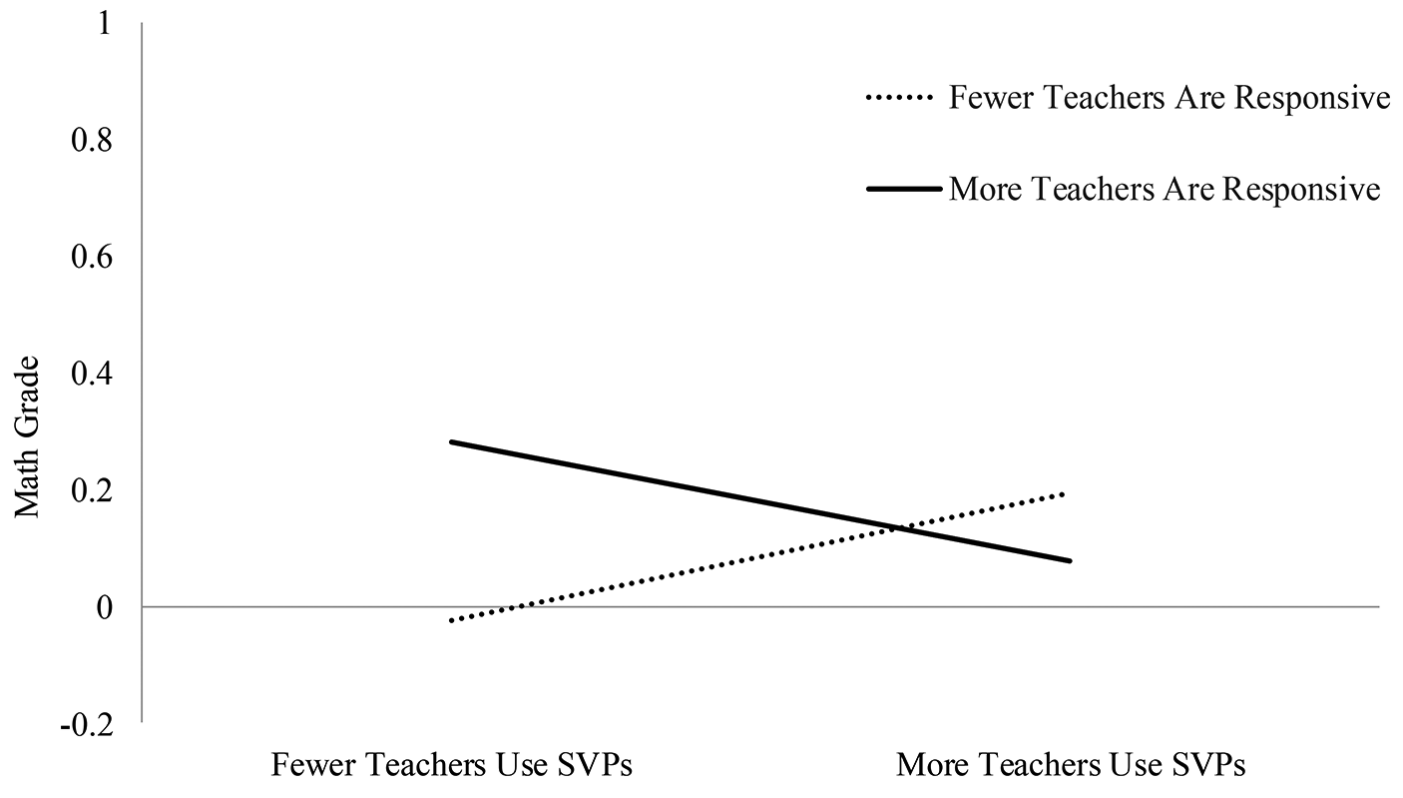
Simple slope analyses of the interactions between SVPs and teacher responsiveness on student self-report math grade

The interaction term between linear classroom SVPs and teacher responsiveness was significantly associated with self-reported math grade (β = − 0.11, *SE* = 0.05, *p* < .05). Figure 4 shows that the benefit of teacher responsiveness on self-reported math grades was limited to students who reported fewer teachers using SVPs. This pattern was confirmed by examining simple slopes. A greater likelihood of teachers being responsive to student ideas was related to significantly higher math grades for those students reporting fewer teachers using SVPs (simple slope at 1 *SD* below the mean: β = 0.17, *SE* = 0.08, *p* < .05), but there was no statistically significant impact among students who reported more teachersusing SVPs (simple slope at 1 *SD* above the mean: β = − 0.10, *SE* = 0.06, *p* = ns).

**Fig. 5:**
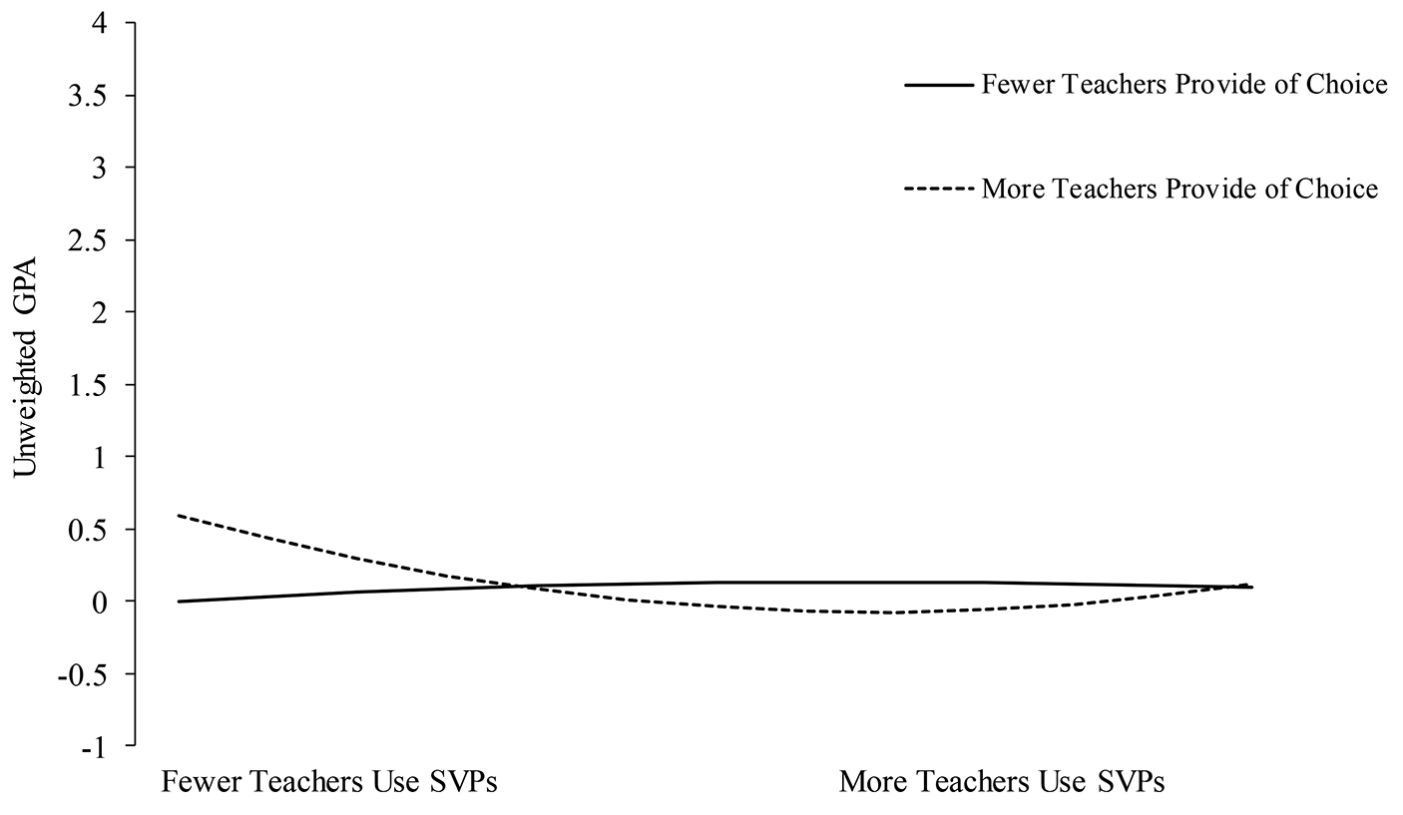
Simple slope analyses of the interaction between quadratic SVPs and teacher provision of choice on student GPA

The interaction term between quadratic classroom SVPs and teacher’s provision of choice was significantly associated with student’s GPAs (β = 0.16, *SE* = 0.07, *p* < .05). Figure 5 shows there is a curvilinear relationship. Among students reporting more teachers providing choice, students appear to report greater GPA when also reporting fewer teachers using SVPs. Among students reporting fewer teachers providing choice, the use of classroom SVPs appears to have little impact on student GPA.

## Discussion

The current study assessed both the linear and curvilinear relationship between SVPs and student outcomes. Although a great deal of prior research has asserted that SVPs improve student engagement and learning (Anderson, 2018; Beattie & Rich, 2018; Scarparolo & Mackinnon, 2022; Toshalis & Nakkula, 2012; Voight & Velez, 2018; Zeldin et al., 2018), few quantitative studies have examined these associations, particularly at the classroom level. Focusing on one specific set of classroom SVPs, the seeking of students’ input and feedback, this study found a strong association between how many of their teachers utilized these strategies and several student outcomes, including academic engagement, student voice agency, absent rates, and ELA grades. We also investigated how teachers offering choice to students in the classroom, showing receptivity to students’ ideas, and demonstrating responsiveness to student input and feedback affected student outcomes, either directly or as moderators of the relationship between classroom SVPs and outcomes. This study advances our understanding of the impact of SVPs in the classroom in several ways.

First, findings reveal that the SVP of seeking students’ input and feedback is significantly associated with greater engagement. Consistent with our hypothesis, the more of their teachers use this practice, the greater levels of engagement students report. This robust finding held up in ANOVAs comparing students who reported different numbers of teachers using the practice and in linear regressions, even when other covariates, such as receptivity, responsiveness, and choice were included in the model. This finding is consistent with other work that shows linkages between SVPs and student engagement (Anderson, 2018; Conner et al., 2022; Voight & Velez, 2018; Zeldin et al., 2018) and buttresses claims about the value of SVPs in promoting students’ engagement in classwork and schoolwork. Teachers and administrators interested in bolstering student engagement might want to pursue professional learning related to this SVP.

In both the ANOVA and regression models, the SVP was also linked to greater student agency, again in line with our hypothesis. Students who experienced more teachers seeking their input and feedback in the classroom were more likely to demonstrate agency in school by expressing concerns and ideas for school improvement. This finding supports a great deal of prior research on the powerful impact of SVPs on student agency (Laux, 2018; Mercer-Mapstone et al., 2017) and suggests that experience with SVPs in the classroom can help build students’ confidence and will to engage in student voice at the school level.

Affirming our hypothesis, the SVP was also associated with lower absent rates in the ANOVA models and in the regressions; however, the effects faded once the other covariates were included in the regression model. Prior work has found that *responsiveness* to student voice *at the school level* is related to better attendance (Kahne et al., 2022). Looking at the individual, rather than the school, we similarly found responsiveness was significantly associated with lower student absent rates in the final model. Together, these results suggest that encouraging teachers to seek their students’ input and feedback *and* to take action in response may be an effective way to improve student attendance and reduce absenteeism.

Departing from our hypotheses, but similar to the mixed findings in prior research, our findings suggest a complicated relationship between classroom SVPs and grades. At the school level, Kahne and colleagues (2022) found that perceived responsiveness to student voice was associated with higher grades; however, looking at the student level, Voight and Velez (2018) found no relationship between student participation in student voice programming and improved academic outcomes. Although we examined teacher practices, rather than school-level SVPs, our study similarly turns up mixed results. While we did find a significant linear association with ELA grades as we expected, we did not find a linear relationship between SVPs and students’ math grades or GPA in the regression models. The more of their teachers sought their input and feedback, the higher ELA grades students reported. Perhaps, the practice of articulating their ideas, concerns, needs and preferences to teachers supports the development of skills valued in the ELA classroom. Existing research has found that engagement in SVPs strengthens students’ communication skills (Bahou, 2012; Keogh & Whyte, 2005).

Although the prevalence or paucity of teachers using the SVP of input and feedback did not appear to be associated linearly with either math grades or GPA, the quadratic and ANOVA findings did point to some interesting patterns. In the regression models, the quadratic relationship between SVPs and GPA was significant and concave, with students initially reporting higher GPAs as more teachers use SVPs, but the gain decreases as even more teachers use SVPs. This relationship is further illustrated with the ANOVA results, which show that students who indicate that “some” of their teachers seek their input and feedback report significantly higher math grades and earn higher GPAs than students who report “none” of their teachers using this practice. Additionally, those who report having only one teacher use the SVP report significantly higher GPAs than those who have no teachers using the practice. These findings raise the possibility that there may be a “sweet spot” in SVP that is more potent than a saturation effect in influencing student academic performance. It may be that having one teacher or some teachers earnestly seek their input and feedback is more advantageous academically to students than having all their teachers do so in a perfunctory way or none of their teachers do so. These ANOVA findings also raise questions about which teachers are using the practice and how a specific teacher’s use of the practice may affect students’ performance in their specific classes or translate to success in other classes.

The current study extends existing research by investigating the shape of the associations between SVPs and student engagement and academic performance with the inclusion of the quadratic term. Findings suggest that in several instances there is a nonlinear relationship between teachers’ use of classroom SVPs and student outcomes. A curvilinear association between SVPs and student engagement, student voice agency, and GPA were found. There were no significant curvilinear relationships found between SVPs and student absenteeism rate, ELA grades, or math grades. Both the linear and quadratic terms were positive in the student engagement and student voice agency models suggesting a convex relationship, where the gain in engagement and agency increases with more teachers using SVPs. In contrast, the relationship between SVPs and GPA was concave, showing that the impact of having teachers use classroom SVPs is impactful to a point and then declines. These findings demonstrate that the relationship between SVPs and student outcomes may not always be linear and that more investigation is needed to understand the level of SVPs that yields the most positive impact on student outcomes.

Across all our analyses, teacher receptivity proved particularly powerful. It was associated with all outcome variables: academic engagement, student voice agency, absent rate, ELA grades, Math grades, and GPA, even when other practices were included in the models. The importance of this aspect of a teacher’s practice, therefore, cannot be understated. More than the SVP of seeking students’ input and feedback, more than showing responsiveness to their input and feedback, and more than offering choice to students, teacher receptivity was consistently and strongly related to all student outcomes.

The significance of receptivity raises implications for teachers as well as future research. Signaling receptivity by simply expressing a desire to hear students’ concerns and ideas for improving the classroom may be one way that teachers can promote student engagement, agency, and achievement without necessarily having to dedicate class time or homework assignments to seeking their feedback and input. More longitudinal research, however, is needed to test the directionality of these relationships over time. It could well be the case that students who are faring well academically are more likely to perceive their teachers as receptive to their ideas and critiques than students who are struggling academically. In-depth qualitative research could also explore the discernment process students go through to assess teacher receptivity. How perceptions of teacher receptivity to student voice relate to the strength of the student-teacher relationships that students report is another fertile area for future research.

Contrary to our hypotheses, this study found no interaction effect with receptivity, suggesting that perceptions of receptivity neither strengthen nor attenuate the relationship between classroom SVPs and student outcomes. Instead, receptivity is the prime driver of outcomes.

The lack of linear interaction effects with choice did not support our hypothesis that coupling choice practices with classroom SVPs would strengthen student outcomes. Instead, we found that the curvilinear relationship between SVPs and engagement and GPA varied by teachers’ provision of choice. When fewer teachers offer choice, having more teachers use SVPs was associated with stronger engagement but had no bearing on GPA. GPA is higher, however, when students reported few teachers using SVPs and more offering choice. These findings present a puzzle and raise questions about the extent to which students perceive choice and SVPs as distinctive practices. Future research could use multigroup modeling to discern whether the interaction effect on GPA between choice and this SVP holds up with students in different ethno-racial groups and different academic tracks. Additionally, there is a need for longitudinal data to assess whether the greater engagement that is associated with this SVP over time leads to greater academic performance.

Several linear and quadratic interaction effects were found between responsiveness and classroom SVPs. When fewer teachers use the SVP of seeking students’ input and feedback, students who rate those few teachers as more responsive to their voices report greater engagement, more student voice agency, and higher math grades than do those with less responsive teachers. Given the literature that shows how demoralizing it can be to students when teachers or school administrators seek their perspectives only to disregard them (Hipolito-Delgado, 2023; Salisbury et al., 2020), it makes sense that students would be more engaged when the few teachers who use SVPs are more responsive to their feedback and input. It also makes sense that in a context where few teachers use SVPs, having more responsive teachers would help promote students’ sense of agency and willingness to speak up to share their concerns and ideas. SVPs coupled with responsiveness can facilitate a virtuous cycle with student voice agency, wherein the practices strengthen one another. The interaction effect of responsiveness and SVPs on math grades is more curious. Why the benefits of responsiveness to students’ math grades fade when more teachers seek their input is unclear; future research could explore who these few responsive teachers are and how students’ experiences with them may or may not transfer to other classroom contexts.

Although it had several strengths, including a large sample size and the use of both self-report and administrative data for assessing student outcomes, the present study had several limitations. It relied on cross-sectional data, preventing us from making causal claims. It was conducted in one urban district, with a diverse, but heavily Latiné population. As a result, the findings may not be generalizable to other populations. Rather than asking about specific teachers, it asked students how many of their teachers used certain practices, thereby examining the overall effects of students’ experiences with classroom SVPs on student outcomes instead of a more narrowly tailored effect, such as that of their math teachers’ SVPs. This makes it difficult to disentangle the impact of a particular teacher’s SVP on student achievement in their classroom. Additionally, because our data were limited to two middle schools and two high schools, and each school may have had unique or eccentric conditions that impacted our results, we were unable to explore school level differences using multigroup modeling.

These limitations signpost new directions for future research to build on and add nuance to the findings of this study. Scholars can pursue longitudinal, mixed methods research, incorporating teachers’ as well as students’ perspectives, in different districts and school types to further examine the relationships explored in this study. There is an acute need for more studies across contexts and communities that attend to the unique ways in which SVPs are experienced by and impact students with different identities, particularly marginalized identities. Researchers can also hone in on one or two specific subject areas and examine how a specific teacher’s use of SVPs relates to their students’ academic achievement in their classroom, adapting the scales used in this study. Math teachers may be of particular interest, given this study’s surprising findings about math grades. Finally, more work exploring how students gauge teacher receptivity and how this construct relates to their experiences in school is warranted.

## Conclusion

This study is the first to examine quantitatively the linear and curvilinear academic impacts of a specific set of classroom SVPs, those that involve seeking students’ input and feedback. Students report higher engagement when more of their teachers use this type of SVP. They also have lower absent rates, higher ELA grades, and show greater agency in school-level SVPs. In addition, when few of their teachers use the practice, but those few are more responsive to students’ input, students report higher math grades. Moreover, better outcomes were achieved when students reported only one or some teachers using the SVP as compared to no teachers using it. While the affordances of this type of SVP are compelling, the strongest across-the-board impacts on student outcomes came from students’ perceptions of teacher receptivity. The more of their teachers they perceived as receptive to their ideas and concerns, the better engagement, agency, attendance, and achievement outcomes students had. Actively seeking student input and feedback and then showing responsiveness to students’ concerns and ideas are direct ways teachers can signal their receptivity to students and promote their students’ success.

## Footnotes

1 We also measured student self reported overall grades this school year. In the regression models, the results for this dependent variable were no different than the results for unweighted GPA, affirming other research that indicates students can be reliable reporters of their grades (Wigfield & Wagner, 2005). In the ANOVA models we did find a significant difference in mean self-reported overall grades, such that students who reported at least one teacher used classroom SVPs (M = 4.01; SD = 1.05) reported higher overall grades than students who reported none of their teachers used classroom SVPs (M = 3.76; SD = 1.18).
